# The Effects of Donor-Recipient Age and Sex Compatibility in the Outcomes of Deep Anterior Lamellar Keratoplasties

**DOI:** 10.3389/fmed.2021.801472

**Published:** 2022-01-27

**Authors:** Hon Shing Ong, Nathalie Chiam, Hla Myint Htoon, Ashish Kumar, Anshu Arundhati, Jodhbir S. Mehta

**Affiliations:** ^1^Corneal and External Diseases Department, Singapore National Eye Centre, Singapore, Singapore; ^2^Singapore Eye Research Institute, Singapore, Singapore; ^3^Duke-NUS Medical School, Singapore, Singapore; ^4^School of Material Science and Engineering, Nanyang Technological University, Singapore, Singapore

**Keywords:** corneal transplantation, keratoplasty, eye banking, anterior lamellar keratoplasty, HLA compatibility, graft rejection, graft survival, graft failure

## Abstract

**Purpose:**

Corneal transplantations are the commonest allogenic transplant surgeries performed worldwide. Transplantable grade donor cornea is a finite resource. There is thus an impetus for eye banks to optimize the use of each harvested cornea, and clinicians to minimize the risks of graft rejection and failure. With better survival and lower rejection rates, anterior lamellar keratoplasty has gained popularity as an alternative technique to full-thickness penetrating keratoplasty, for the treatment of corneal stromal diseases. This study evaluated the effects of donor-recipient age- and sex-matching on the outcomes of eyes that had undergone deep anterior lamellar keratoplasty (DALK) surgeries.

**Design:**

Observational cross-sectional study (national corneal graft registry data).

**Subjects:**

All DALK surgeries performed in a tertiary ophthalmic hospital over an 11-year period.

**Methods:**

To analyse the effects of donor-recipient sex-matching, transplantations were classified as “presumed H-Y incompatible” (male donor to female recipient) or “presumed H-Y compatible” (all other donor-recipient sex combinations). For age-matching, differences in donor and recipient ages were calculated. Cox proportional hazards regressions were used to evaluate the influence of donor-recipient sex-matching and age-matching on graft failure and rejection.

**Main Outcome Measures:**

Rates of graft failure and rejection within each group.

**Results:**

401 eyes were included. 271 (67.6%) transplants were presumed H-Y compatible. 29 (7.2%) grafts failed and 9 (2.2%) grafts rejected. There were trends of lower hazard ratios (HRs) in graft failure and rejection in the presumed H-Y compatible group [HRs: 0.59 (95% CI 0.20–1.77, *p* = 0.34) and 0.93 (95% CI 0.22–3.89, *p* = 0.926), respectively]. Median difference in age between recipients and donors was 15.0 years (IQR −2.8–34.3). The HRs of graft failure and rejection were not influenced by donor-recipient age [HRs per 1-year increase in age difference: 0.995 (95% CI 0.98–1.01, *p* = 0.483) and 1.01 (95% CI 0.99–1.03, *p* = 0.394), respectively].

**Conclusion:**

In eyes that had undergone DALK surgeries, no significant influence of donor-recipient sex- or age-matching on graft rejection and failure was observed. Without strong evidence and the limitations of obtaining sample sizes required for an adequately powered study, the benefits of sex- and age-matching of donors and recipients during graft allocation for DALK surgeries is currently inconclusive.

## Introduction

Corneal transplantations are the most common allogenic transplant surgeries performed worldwide (1, 2). A global report indicated that in 2012, a total of 283 530 donor corneas were harvested annually and stored in 742 eye banks worldwide (3). Of these, 184,576 were used to perform corneal transplantation surgeries in 116 countries (3). Similarly, the Eye Bank Association of America reported that in 2018, of the 133,576 donor corneas procured by 57 eye banks in the United States, 85,441 were used to perform transplantation surgeries (2). As the cornea is thought to be immunologically privileged, (4) compared to other forms of solid organ transplantations, corneal tissue allografts are associated with comparatively lower risks of immunological rejection and less requirements for prolonged systemic immunosuppression (5–11).

Penetrating keratoplasty (PKP) is a full-thickness corneal transplantation technique where all layers of the corneas are replaced. Since its introduction in 1905, for more than a century, PKP has been the predominant procedure for the treatment of visual loss from corneal diseases. Over the past two decades however, there has been a paradigm shift in the surgical treatment of corneal diseases to perform selective tissue transplantation i.e., anterior lamellar keratoplasty (ALK) or endothelial keratoplasty (EK), where only diseased layers of the cornea are replaced (2, 9, 12–14). In ALK, the anterior diseased layers of the recipient's cornea are replaced (15); in EK, a posterior lamellar keratoplasty technique, the diseased corneal endothelium of the recipient is replaced (16). These two “lamellar” keratoplasty techniques, have been shown to achieve lower risks of immunological graft rejection and improved graft survival rates (9, 17–22). With these advantages, lamellar keratoplasties have thus gradually been adopted as preferred corneal transplantation techniques in various institutions (2, 9, 13). Nevertheless, graft rejection and failure do occur following all types of corneal transplantations (23, 24).

With only one in 70 of the global demands of corneal transplantations being met, there is a shortage of suitable donor corneas (3). Indeed, transplantable grade donor corneal tissue is a finite and scarce resource. There is therefore a strong impetus for eye banks to optimize the use of each harvested donor corneal tissue, and clinicians to minimize the risks of graft rejection and failure. Attempts to lower the risks of graft rejection can begin pre-operatively during donor tissue allocation. In allogeneic solid organ transplantations, donor-recipient human major histocompatibility antigens / human leukocyte antigens (HLA) matching is one of the most important considerations in donor tissue allocations. This is particularly important for kidney and bone marrow transplantations (25). On the contrary, HLA matching is not routinely performed in corneal transplantations, as existing evidence suggests that this practice does not confer a significant graft survival benefit (23, 26–29). Some studies have, however, investigated the effects of donor-recipient age- or sex-matching in corneal graft allocations (30–34). In age-matching, recipients are assigned grafts from donors who are of the same age group. This is commonly practiced in many eye banks, (30, 35) despite a lack of evidence on whether grafts from older donors perform as well as grafts from younger donors in young recipients, and vice versa (30). In sex-matching, donor corneal tissues are allocated to recipients of the same sex (32, 34, 36). Additionally, a further subtype of sex-matching is H-Y compatibility. The H-Y antigen, which is HLA-A1 restricted, is expressed by the Y chromosome and is found in A1-positive males (36–39). H-Y incompatible grafts refer to grafts from A1-positive male donors being transplanted into female recipients or into A1-negative male recipients, whilst H-Y compatible grafts refer to the other possible donor-recipient combinations (i.e., A1-positive male donor grafts to A1-positive male recipients, female donor grafts to female or male recipients).

Published literature on donor-recipient age or sex compatibility has thus far mainly been focused on the outcomes of PKP procedures (31, 34, 36, 40–43). Studies on PKP have reported beneficial effects of sex or H-Y matching in lowering the risks of graft rejection and improving graft survival (31, 34, 36). Nevertheless, the current evidence for donor-recipient sex-matching is still considered equivocal, as several other studies have failed to show significant benefit in sex- or H-Y antigen matching (40, 42, 43). Such varying results have been attributed to differences in study designs, diversities in ethnic populations, or inadequate sample sizes (31, 34, 36, 40, 42, 43). With the shift away from performing PKP procedures, investigators have set out to evaluate the effects of sex-matching in eyes undergoing lamellar keratoplasties, in particular, EKs (32, 33). However, the evidence of donor-recipient sex-matching in EK has also so far been inconsistent, (32–34) with some studies observing lower rates of graft survival with male donors (33) but others failing to show similar associations (32).

Anterior lamellar keratoplasty (ALK), the other selective tissue transplant procedure, has gained popularity as an alternative to PKP to treat corneal stromal diseases (15, 17, 44). Examples of such diseases include keratoconus, corneal dystrophies, or stromal scars caused by a variety of insults (e.g., infection, trauma). In ALK, the recipient's own Descemet membrane (DM) and corneal endothelium are retained. As endothelial graft rejection is the most commonly encountered form of immunological rejection following corneal transplantations, the observed rates of graft rejection in ALK are therefore significantly lower compared to PKP or EK (9, 19, 22, 44). Despite being less common, immunological rejections, namely epithelial, subepithelial, or stromal rejections, still do occur following ALK (22). Stromal rejection rates after ALK have been reported to be ~5% but can be as high as 25% within the first 18 months following transplantation (45–47). Nonetheless, the significance of donor-recipient age- or sex- matching specifically for ALK surgery, has yet to be explored. Using data from the Singapore national transplant registry, we evaluated the effects of donor-recipient age- and sex- matching on the surgical outcomes of eyes that had undergone deep anterior lamellar keratoplasty (DALK) surgeries.

## Methods

### Study Population

This was a cross-sectional analysis of consecutive DALK surgeries performed in the Singapore National Eye Center from 2004 to 2015. Data was obtained from the Singapore Corneal Transplant Study (SCTS) database. This national transplant registry database is maintained by the Singapore Eye Bank where the surgical outcomes of all corneal transplants performed in Singapore are prospectively collected. The study protocol adhered to the tenets of the Declaration of Helsinki and is approved by the SingHealth Centralized Institutional Review Board (CIRB reference: 2018/2688).

The Singapore National Eye Center is a tertiary referral center that performs approximately 80% of corneal transplants in Singapore. The DALK surgeries were performed by a group of corneal surgeons in our center, including trained corneal specialists and corneal fellows-in-training. All surgeons used the same techniques of DALK. DALK surgeries were performed using either the modified big bubble technique or a pre-Descemetic manual layer-by-layer dissection technique (48). Post-operatively, topical steroids (*Guttae* prednisolone acetate 1% or Dexamethasone 0.1%) together with topical fluoroquinolone antibiotics (*Guttae* levofloxacin 0.5% or moxifloxacin 0.5%) were administered at 3-hourly intervals and gradually tapered over 6–8 months. Sutures were removed between post-operative month 4–18, guided by visual acuities and corneal astigmatism.

As is standard practice at the Singapore Eye Bank, donor-recipient ages were matched as closely as possible, where recipients were allocated corneal tissues from donors of approximately the same age groups. No donor-recipient matching for sex was performed in the allocation of corneal grafts to the recipients. In our study, we only included cases of DALKs performed for optical indications. As DALKs that are performed for tectonic or therapeutic reasons tend to have poorer outcomes compared to grafts performed for optical indications, (49) these cases were excluded to avoid adding unnecessary heterogeneity into the data. For eyes that had undergone repeat grafts, the outcome data of the first (primary) corneal grafting of these eyes were analyzed.

### Data Collected and Primary Outcomes

Pre-operative variables collected included age and sex of both recipients and donors. Recipient eye characteristics collected included indications for transplant surgeries, co-existing ocular diseases, and the presence of risk factors for graft failure or rejection (such as cornea vascularisation, ocular surface disease, active ocular inflammation, and glaucoma). High-risk grafts were defined as cases with at least one of the aforementioned risk factors for graft failure or rejection. Post-operative data collected included the presence and recorded date of occurrence of graft rejection, graft failure, and any other post-operative complications. From this data, the duration of rejection-free period and graft survival were extrapolated. Graft rejection was defined as corneal oedema and the presence of epithelial or stromal inflammation in a graft that was previously clear (50). Graft failure was defined as persistent graft oedema that compromised vision for a minimum of three consecutive months (50).

### Statistical Analysis

All statistical analyses were performed using Stata version 15.0 (StataCorp, College Station, Texas, USA). The unit of analysis was outcomes for eyes. To account for the cluster effect of within-patient inter-eye correlations in patients who had DALK surgeries performed in both eyes, cluster-correlated analysis was performed. The primary outcomes were graft rejection and graft failure. To analyse the effects of donor-recipient age-matching on graft rejection and failure, the donor-recipient age gap was calculated. Donor age gap = age of recipient – age of donor in years. To analyse the effects of donor-recipient sex-matching and presumed H-Y compatibility on graft rejection and failure, we looked at presumed H-Y incompatible grafts [male donor to female recipient (M-F)] and compared them to presumed H-Y compatible grafts [male donor to male recipient (M-M), female donor to male recipient (F-M), female donor to female recipient (F-F)]. The term “presumed compatibility” was used as the H-Y antigen is HLA-A1 restricted; it is only expressed by the Y-chromosome in A1-positive males (36–39) and the frequencies of the A1 allele varies amongst different ethnic populations (51).

Kaplan-Meier curves of graft survival time and rejection-free survival time were generated; the differences between presumed H-Y compatible and incompatible groups were also assessed with log-rank tests. Additionally, Cox proportional hazards regression were used to evaluate the influence of age- and sex-matching on the hazards of graft rejection and failure. Univariable and multivariable analyses were also conducted. The multivariable cox regression model was adjusted for donor age, recipient age, donor sex, recipient sex, indications of graft, and whether the graft was considered low or high risk. Using the observed rates of events and hazard ratios determined in this study, Cox regression power analyses were used to estimate the sample sizes required to achieve sufficient power (80%) to show a significant difference if any, in rates of graft survival and graft rejection. A *p* < 0.05 was considered statistically significant.

## Results

A total of 540 DALK surgeries were performed over the 11-year study period. Of these, 56 surgeries were performed for tectonic or therapeutic indications and were excluded. Of the 484 surgeries performed for optical indications, 24 were repeat transplants; no follow-up data were available in 59 cases. Thus, a total of 401 eyes that underwent DALK surgeries for optical indications were included in our analyses. The characteristics of our donor and recipient population is reported in Table 1. The most common indications for DALKs were keratoconus (*n* = 167, 41.6%), corneal dystrophies (*n* = 42, 10.5%), cornea scar from infective keratitis (*n* = 50, 12.5%), and cornea scar from interstitial keratitis (*n* = 22, 5.5%). The remaining cases were performed for less common indications such as corneal scar from chemical injuries or ocular surface diseases. Amongst the recipients, the median age was 33.3 (IQR: 22.8–53.9) years and 192 (47.9%) were female. Amongst the donors, the median age was 55.0 (IQR: 40.0–66.0) years and 126 (31.4%) were female. The median difference in age between recipients and donors was 15.0 years (IQR: −2.8–34.3). With regard to donor-recipient sex-matching, 130 (32.4%), 145 (36.2%), 64 (15.9%), and 62 (15.5%) were M-F, M-M, F-M, and F-F, respectively. Ninety-six grafts (23.9%) were classified as high risk grafts.

**Table 1:** Descriptive data of both donors and recipients.

	**Entire study population**	**Presumed HY Incompatible (M-F)**	**Presumed HY compatible (M-M, F-M, F-F)**
Number of eyes	401	130	271
Graft failure *n* (%)	29 (7.2)	11 (8.5)	18 (6.6)
Graft rejection *n* (%)	9 (2.2)	3 (2.3)	6 (2.2)
**Donor demographic characteristics** ^*^			
Age, years median (IQR)	55 (40–66)	53 (36–63)	56 (42–68)
Female gender, *n* (%)	126 (31.4)	–	–
Race, *n* (%)	389	128	261
Chinese	73 (18.8)	24 (18.8)	49 (18.8)
Malay	7 (1.8)	2(1.6)	5 (1.9)
Indian	46 (11.8)	18 (14)	35 (13.4)
Others	263 (67.6)	84 (65.6)	172 (65.9)
Recipient demographic characteristics^†^^*^			
Age, years median (IQR)	33.3 (22.8–53.9)	35.6 (23.5–55.7)	32.9 (22.5–53)
Female gender, *n* (%)	192 (47.9)	–	–
Race, *n* (%)			
Chinese	148 (36.9)	52 (40)	96 (35.4)
Malay	57 (14.2)	20 (15.4)	37 (13.7)
Indian	73 (18.2)	21 (16.1)	52 (19.2)
Others	123 (30.7)	37 (28.5)	86 (31.7)
**Recipient ocular characteristics**^†^ **Low or high-risk graft**, ***n*** **(%)**			
Low risk	305 (76.1)	97 (74.6)	208 (76.8)
High risk	96 (23.9)	33 (25.4)	63 (23.2)
**Optical indication for corneal transplant**, ***n*** **(%)**			
Keratoconus	167 (41.6)	47 (36.2)	120 (44.3)
Corneal dystrophies	42 (10.5)	15 (11.5)	27 (10.0)
Scar from previous infective keratitis	50 (12.5)	22 (16.9)	28 (10.3)
Scar from previous interstitial keratitis	22 (5.5)	7 (5.4)	15 (5.5)
Miscellaneous (e.g., scars from other pathologies)	120 (29.9)	39 (30.0)	81 (29.9)
**Donor-recipient matching characteristics** ^†^			
**Donor-recipient sex matching (4 groups)**, ***n*** **(%)**			
Male to Female (M-F)	130 (32.4)	130 (100.0)	0
Male to Male (M-M)	145 (36.1)	0	145 (53.5)
Female to Male (F-M)	64 (16.0)	0	64 (23.6)
Female to Female (F-F)	62 (15.5)	0	62 (22.9)
Difference in donor vs. recipient age	15.0 (−2.8–34.3)	10.8 (−6.9–30.1)	17.3 (0.14–35.5)

* *Data are presented as median (interquartile range, IQR) or n (%) as appropriate.*

† *Eye-specific variables*.

### Effects of Sex-Matching on Graft Survival

**Figure 1:**
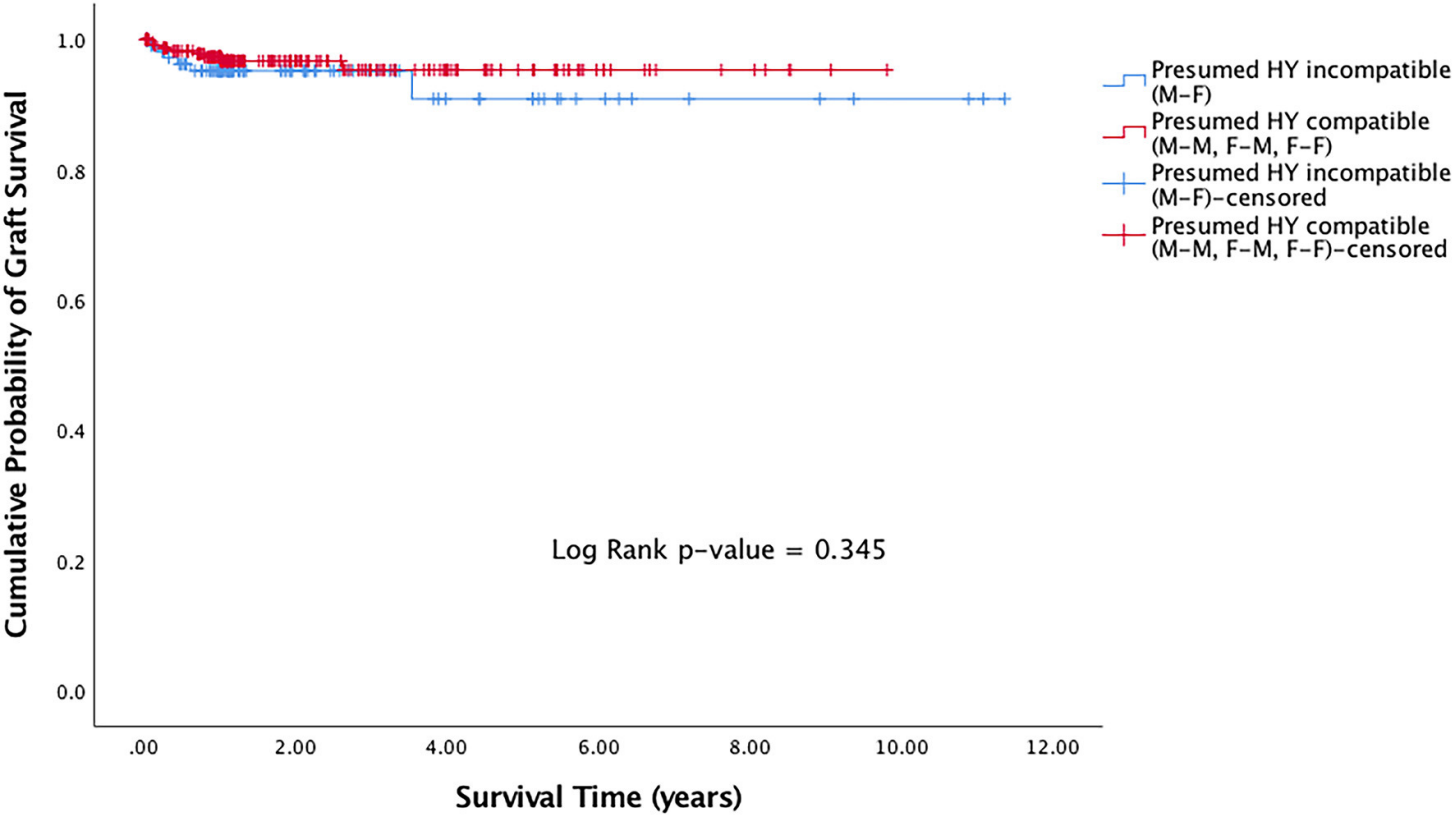
Kaplan Meier graft survival curve for presumed H-Y incompatible and H-Y compatible grafts.

29 (7.2%) of DALK grafts had failed. Of the grafts that failed, the median time to graft failure was 0.38 years (IQR 0.15–0.84). Presumed H-Y incompatible grafts (M-F grafts) showed a trend of worse survival compared to presumed H-Y compatible grafts (M-M, F-M, F-F), although this was not statistically significant (*p* = 0.345) (Figure 1).

### Effects of Sex-Matching on Graft Rejection

**Figure 2:**
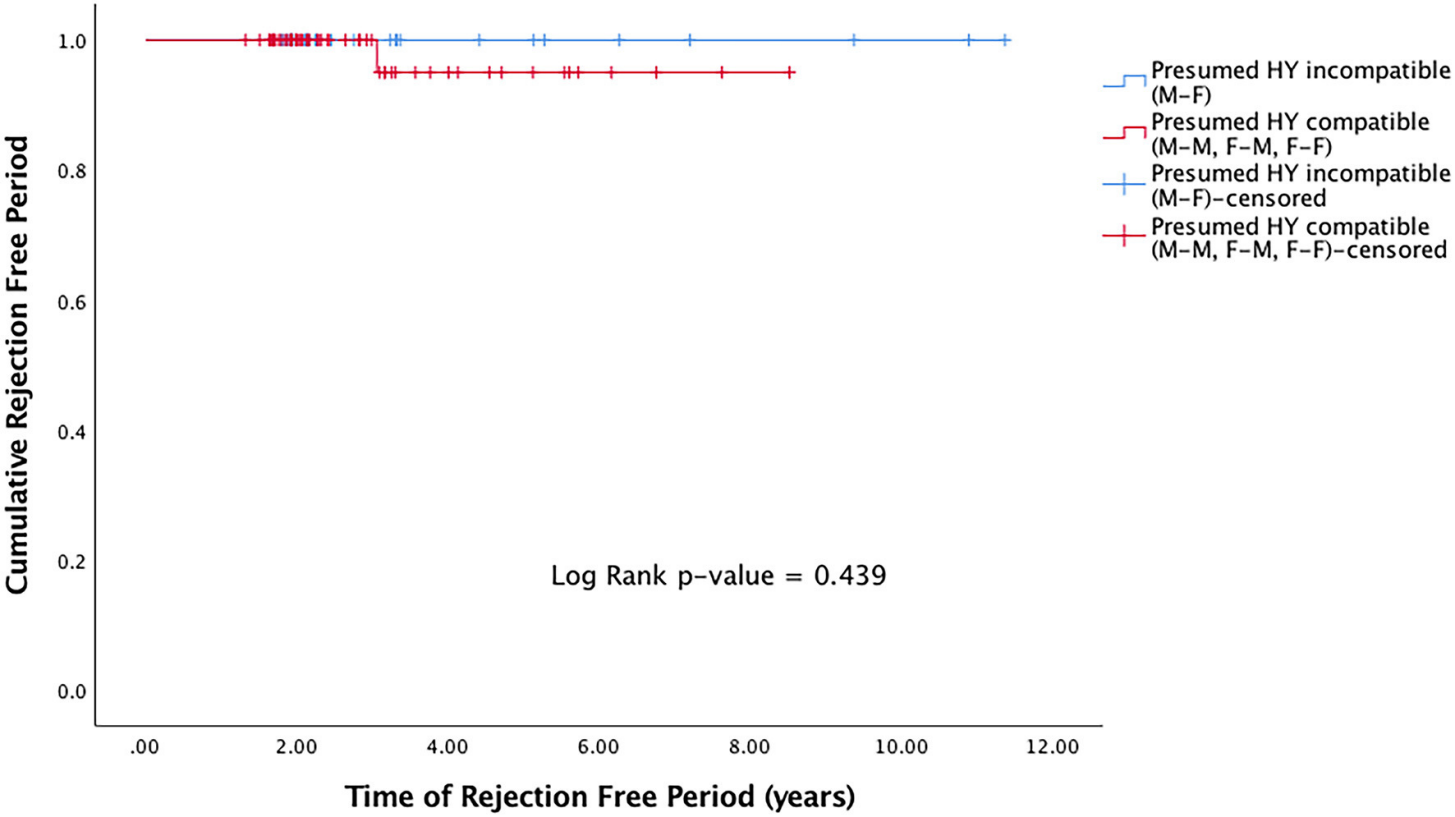
Kaplan Meier rejection-free survival curve for presumed H-Y incompatible and H-Y compatible grafts.

Rejection episodes were recorded in 9 (2.2%) grafts. Of the grafts that suffered rejection, the median time to rejection was 1.1 years (IQR 0.93–3.0). There was no significant difference in rejection-free duration in presumed H-Y incompatible grafts (M-F grafts) compared to presumed H-Y compatible grafts (M-M, F-M, F-F) (*p* = 0.439) (Figure 2).

Table 2 presents the hazard ratios (HR) of graft failure and rejection in our analyses of donor-recipient sex-matching. The HRs of graft failure were lower in presumed H-Y compatible grafts (M-M, F-M, F-F grafts) compared to presumed H-Y incompatible grafts (M-F grafts), although this was not statistically significant in both univariable and multivariable analyses (*p* = 0.329 and *p* = 0.347, respectively). Similarly, there were trends that the HRs of graft rejection were lower in presumed H-Y compatible grafts (M-M, F-M, F-F grafts) compared to presumed H-Y incompatible grafts (M-F grafts), although this was not statistically significant in both univariable and multivariable analyses (*p* = 0.842 and *p* = 0.926, respectively). Table 3 presents the HR of graft failure and rejection in our analyses of donor-recipient age-matching. The donor-recipient differences in age did not have a significant influence on graft failure or rejection in both univariable and multivariable analyses.

**Table 2:** Associations of donor-recipient sex-matching with graft failure and rejection.

	**Total**	**Events**	**Univariable model**			**Multivariable model^a^**		
	**N**	**N (%)**	**HR**	**95% CI**	**P**	**HR**	**95% CI**	**P**
**Graft failure**								
**Donor-recipient sex-matching**								
Presumed HY-incompatible	130	11 (8.5)	Reference	–	–	Reference	–	–
Presumed HY-compatible	271	18 (6.6)	0.59	0.21–1.69	0.329	0.59	0.20–1.77	0.347
**Graft rejection**								
**Donor-recipient sex-matching**								
Presumed HY-incompatible	130	3 (2.3)	Reference	–	–	Reference	–	–
Presumed HY-compatible	271	6 (2.2)	0.87	0.21–3.56	0.842	0.93	0.22–3.89	0.926

*HR, hazards ratio; CI, confidence interval.*

a *Multivariable model adjusts for high risk graft, indications of graft, age of donor, and age of recipient*.

**Table 3:** Associations of donor-recipient age-matching with graft failure and rejection.

	**Total**	**Events**	**Univariable model**			**Multivariable model^a^**		
	**N**	**N (%)**	**HR**	**95% CI**	**P**	**HR**	**95% CI**	**P**
**Graft failure**								
Difference in donor vs. recipient age, per 1-year increase	401	29 (7.2)	0.994	0.98–1.01	0.453	0.995	0.98–1.01	0.483
**Graft rejection**								
Difference in donor vs. recipient age, per 1-year increase	401	9 (4.2)	1.01	0.99–1.02	0.302	1.01	0.99–1.03	0.394

*HR, hazards ratio; CI, confidence interval.*

a *Multivariable model adjusts for high risk graft, indications of graft, sex of donor, and sex of recipient*.

## Discussion

In this study, we evaluated the effects of donor-recipient sex- and age-matching on the risks of graft failure and rejection in eyes that had undergone DALK surgeries. In our cohort of 401 consecutive DALK grafts performed over an 11-year period (2004–2015), there were trends of improved graft survival in presumed H-Y compatible grafts (Figure 1). Through univariable and multivariable regression models on risks of graft rejection and failure, we also showed trends of a protective effect when presumed H-Y compatible grafts were used (Table 2). Overall however, our results did not achieve statistical significance. When we evaluated donor-recipient age-matching in our series, such trends seen in sex-matching on graft rejection and survival, were not observed.

A potential graft survival benefit of donor-recipient sex-matching was first reported by Völker-Dieben et al. (31). Reporting on the clinical outcomes of 539 PKP procedures, the authors observed that female donor corneas had significantly better one-year graft survival in female recipients compared to male recipients (31). More recently, interest in the concept of sex-matching for corneal transplantations was renewed after two studies independently reported lower rates of graft rejection and failure amongst donor-recipient sex-matched or H-Y antigen compatible transplantations (34, 36). In a series evaluating 229 HLA-A1 donor positive keratoplasties, Bohringer et al. showed a significant benefit in rejection-free graft survival in H-Y compatible transplantations. In a subsequent UK based study which included 18,171 patients who had undergone predominantly PKP procedures, Hopkinson et al. showed that in certain pathologies, such as Fuchs' endothelial dystrophy and keratoconus, patients receiving biological sex-mismatched donor tissues were at greater risk of graft rejection and failure (34).

Not all studies have reported similar beneficial effects of sex-matching (40, 42, 43). Even in the initial 1982 series by Völker-Dieben et al. showing graft survival benefits of female to female transplantations, male donors to male or female recipients did not influence graft survival (31). Furthermore, when the same investigators incorporated a larger number of grafts in their 1987 report, sex-matching no longer had a significant association with graft failure (43). Similarly, a Japanese study of 396 eyes that had undergone PKP procedures found no additional benefit in lowering the risks of rejection with sex-matching (42). In another study based on a Korean population, their presumed H-Y compatible group was not associated with improved rejection-free PKP graft survival (40).

The evidence of sex-matching in lamellar keratoplasties, is even much less clear. In 2017, a Swedish based study investigating 1,789 EK procedures showed that male donor sex was associated with lower rates of graft survival in a univariate regression model (33). Although the authors explained their finding through immunological mechanisms, they believed that other undetermined mechanisms may also be involved (33). Studies have reported that adrenaline can affect corneal endothelial function (52, 53). Unintentional injuries (which includes trauma from falls, burns, road traffic accidents etc.) are associated higher systemic release of adrenaline, and account for more male deaths (7.6%) (54) compared to female deaths (4.4%) (55); this could thus potentially explain the lower EK graft survival rates observed with male donors (33). Nevertheless, when covariate models were applied in the Swedish study, sex-matching failed to be predictive of graft failure (33). Furthermore, donor sex was not associated with risks of graft rejection (33). Similarly, in a 2018 study reporting outcomes of over 2,000 EK procedures, the investigators failed to show significant effects of reducing graft rejection and improving graft survival in sex-matched transplantations (32). Our study, being the first to investigate the effects on anterior lamellar procedures alone, similarly supports the other studies on EK, in the notion that in lamellar surgery, sex-mismatching is less of a concern.

Several factors may explain why the beneficial effects of sex-matching have been observed in studies involving PKP but not in studies of lamellar keratoplasties, such as ours. It has been well-reported that the risks of graft rejection and failure, are significantly lower in lamellar keratoplasty procedures compared to full-thickness PKPs (9, 17–22). Depending on the pre-operative diagnoses for performing PKP, five-year graft survival rates have been reported to be as low as 21% and graft rejection rates to be as high as 68% in some series (56, 57). As a result of higher incidences of graft rejections and failures in PKP procedures, the effects of sex-matching are therefore more likely to be observed. In our study of DALK surgeries, the rates of graft failure and rejection were low at 7.2 and 2.2%, respectively. A much larger sample size would thus be required to show a beneficial effect, if any, of sex-matching in lamellar keratoplasties. It would indeed be beyond the practicality of most institutions to obtain the required number of lamellar transplantations to show statistical significance. To illustrate this, with our current observed rates of events and hazard ratios (Table 2), using Cox regression power analyses (α-level = 0.05), the numbers of DALK surgeries required for the study to have 80% power to show significant effects of sex-matching (aiming to detect a 50% difference between the groups), if any, on rates of graft survival and graft rejection are 25,951 and 596,034, respectively (58). Obtaining such numbers of performed DALK surgeries would not be possible, even when multicentred graft registry data are used (7, 34). This limitation was also observed in the large UK-based study using national registry data reported by Hopkinson et al. (34). Although authors demonstrated that the protective effects of H-Y antigen compatibility were significant for PKPs, they acknowledged that the benefits of donor-recipient matching in lamellar keratoplasties (ALK and EK surgeries) were not conclusive due to inadequate numbers (34). From the report however, the overall numbers of lamellar keratoplasties included in their study were unclear.

Another factor explaining the disparity of results between studies evaluating the effects of sex-matched corneal transplantations relates to H-Y compatibility and the prevalence of male individuals who are HLA-A1 positive (31, 34, 36). The H-Y antigen, is expressed by the Y chromosome and is found in HLA-A1 positive male individuals (36–39). All female recipients and A1 negative male recipients may thus theoretically develop an alloimmune response against the H-Y antigens when they receive grafts from A1 positive male donors (59). As H-Y antigens are expressed in A1-positive males, donor-recipient sex-matching has thus been used as a surrogate for H-Y antigen compatibility, as opposed to expensive tests required to detect other major and minor histocompatibility antigens. Indeed, studies have assumed H-Y antigen incompatibility as “all male donor grafts to female recipients”, with all the other possible donor-recipient combinations as H-Y compatible (40, 42). However, this may not be entirely accurate (60). Racial heterogeneity of a population is a confounder when comparing between different datasets. Indeed, the frequencies of the A1 allele varies between different geographical and ethnic populations (51). For example, in our Singapore cohort comprising mostly of patients with Chinese, Malay, and Indian origins, the frequencies of the A1 allele could range between 0.7 and 28.8% (using Singapore-Malaysia allele frequency data), with the prevalence being lower in individuals of Chinese origins and higher in those of Indian origins (51). This may explain why studies investigating donor-recipient sex-matching as a surrogate for presumed H-Y compatibility have failed to show significant effects in graft rejection and survival rates (40, 42, 60). This is especially the case in populations where the frequencies of A1 allele are low (40, 42, 51). Many of the transplants reported in these studies are therefore in fact H-Y compatible.

In addition to sex- or H-Y matching for DALK grafts, we also explored the hypothesis that age-matching may affect graft failure or rejection. It is the practice of many cornea eye banks and surgeons to match donor and recipient grafts for age (24). This practice originated in the early days of penetrating keratoplasties, as it was hypothesized that younger donor grafts had better endothelial cell counts (compared to older donor grafts) and hence should be reserved for younger recipients with longer average life expectancies (61). In our study, we found that older recipient age and older donor age were in fact both associated with lower hazards of graft rejections. However, the difference between donor and recipient age was not associated with the hazards of graft rejection or failure (Table 3). The Cornea Donor study has also investigated the effect of donor-recipient age differences on graft outcomes of penetrating keratoplasty (61). Similarly, they did not observe any adverse graft outcomes when donor-recipient ages were not matched (61). In a study on EK, donor age was also not found to be an independent risk factor for graft survival (33). Thus, there appears to be a lack of evidence of donor-recipient age matching in graft allocation.

Our study has its limitations. Firstly, the retrospective nature of our data limits any inferences of causality, with respect to age- and sex-matching and risks of graft failure and rejection, for which a randomized clinical trial would have served best. Secondly, as mentioned above, because of our low rates of graft failure and rejection, a larger sample size is required to have sufficient power to detect significance differences between matched and unmatched groups. In this study, we used data obtained from the Singapore national transplant registry, where the surgical outcomes of all corneal transplantations performed in Singapore are prospectively collected. This allowed us to obtain data on the 401 DALKs cases performed for optical indications over an 11-year period. As the surgical technique of DALK is challenging and not performed by all corneal surgeons, this large sample size of DALK cases will not be easily replicated by many other institutions. Obtaining the required numbers through other study designs, such as a randomized controlled trial, would not have been possible. Future work may involve combining data from different corneal graft registries to increase the sample size to achieve sufficient statistical power. However, as indicated in our power analyses, obtaining the required number of cases may be difficult even when such registry data are combined.

In conclusion, in our large cohort, in eyes that had undergone DALK surgeries, no significant influence of donor-recipient sex- or age-matching on graft rejection and failure was observed. This adds to the current literature which has thus far been focused on PKP and EK procedures. Without strong evidence and the limitations of obtaining numbers required for an adequately powered study, the benefits of sex- and age-matching of donors and recipients during graft allocation for DALK surgeries remains inconclusive.

## Data Availability Statement

The raw data supporting the conclusions of this article will be made available by the authors, without undue reservation.

## Ethics Statement

This study was reviewed and approved by SingHealth Centralized Institutional Review Board.

## Author Contributions

HO, AA, and JM: conceptualization and supervision. HO, NC, HH, and AK: data curation, formal analysis, investigation, and methodology. HO, NC, and JM: writing draft, review, and editing. All authors approved the manuscript.

## Funding

This work was partially supported by the Lee Foundation SingHealth Transplant Grant (No. SHTX/LFG/001/2019).

## Conflict of Interest

The authors declare that the research was conducted in the absence of any commercial or financial relationships that could be construed as a potential conflict of interest.

## Publisher's Note

All claims expressed in this article are solely those of the authors and do not necessarily represent those of their affiliated organizations, or those of the publisher, the editors and the reviewers. Any product that may be evaluated in this article, or claim that may be made by its manufacturer, is not guaranteed or endorsed by the publisher.
